# On the use of antiangiogenetic medications for retinopathy of prematurity

**DOI:** 10.1111/j.1651-2227.2011.02330.x

**Published:** 2011-08

**Authors:** Anna-Lena Hård, Ann Hellström

**Affiliations:** Department of Ophthalmology, Institute of Neuroscience and Physiology, The Sahlgrenska Academy at University of GothenburgGothenburg, Sweden

**Keywords:** Antiangiogenetic treatment, Anti-VEGF, Propranolol, Retinopathy of prematurity

## Abstract

In contrast to the adult, the third-trimester foetus experiences one of the most intense periods of growth and maturation of its lifetime. Early development is characterized by the existence of critical periods when environmental factors effectively produce long-lasting changes.

Proliferative retinopathy of prematurity (ROP) is a potentially blinding disease characterized by uncontrolled retinal angiogenesis. This pathologic angiogenesis is the target for two new treatment modalities for ROP, i.e. intravitreal anti-VEGF (bevacizumab) and systemic propranolol, which are being evaluated in ongoing or planned studies. VEGF is essential for normal angiogenesis in a growing infant, and the adrenergic system is important for many organ systems and, in addition, for plasticity of the visual and olfactory systems.

**Conclusion:**

This viewpoint raises concerns regarding the currently studied antiangiogenetic treatments for ROP and their possible general effects on the developing preterm infant.

In contrast to the adult, the third-trimester foetus experiences one of the most intense periods of growth and maturation of its lifetime. Early development is characterized by the existence of critical periods when environmental factors effectively produce long-lasting changes. An example is that of the antiangiogenetic substance thalidomide, which during a very limited time period in early pregnancy causes gross malformations. Angiogenesis is important for the alveolarization of the lungs, which in humans mainly takes place after birth in infants born at term (1), and in newborn and infant rats, thalidomide (2) as well as a VEGF-receptor inhibitor (3) reduced lung vascular density and alveolarization. In the central nervous system, maturational processes occur at different times in different brain regions and neural circuits, and therefore, critical periods may be specific for each brain region or neurotransmitter system (4).

The very preterm infant has lost nutrients and other factors supplied by the mother and is exposed to poor nutrition, hyperoxia/hypoxia, infections and other stresses resulting in impaired growth and development. In the eye, reduced physiologic angiogenesis may lead to hypoxia followed by uncontrolled vessel growth. This pathologic angiogenesis is the target for two new treatment modalities for retinopathy of prematurity (ROP), which are being evaluated in ongoing or planned studies. We would like to express our concern about possible adverse effects of these medications on the development of these vulnerable infants.

In the Pan-VEGF Blockade for the Treatment of Retinopathy of Prematurity (BLOCK-ROP) study (ClinicalTrials.gov Identifier: NCT01232777), intravitreal injection of 0.625 or 0.75 bevacizumab (Avastin®), an anti-VEGF antibody, will be compared with standard of care laser for type 1 prethreshold ROP diagnosed at 30–36 postmenstrual weeks. In the Safety and Efficacy of propranolol in newborns with retinopathy of prematurity (PROP-ROP) study (ClinicalTrials.gov Identifier: NCT01079715) (5), preterm infants with stage 2 ROP in zone II or III without plus-disease will receive systemic propranolol, a nonselective beta blocker, up to 90 days in addition to standard care in comparison with standard treatment only.

## Avastin for ROP

VEGF promotes both normal and pathologic angiogenesis, and it is a neuronal survival factor. The blockage of VEGF with Avastin® may thus influence other processes than pathologic angiogenesis in the eye. Bevacizumab is a large molecule, and an advantage put forward is its inability to escape the eye unless in very small amounts (6). However, one intravitreal injection of 1.25 mg/50 μL in three adult cynomolgus macaques weighing 3.9–5.5 kg resulted in a maximum serum concentration of 1430 ± 186 ng/mL 1 week after injection and concentration declined more slowly than in the eye, with little change after 4 weeks, and was 67 ± 24.3 ng/mL after 8 weeks (7).

In a recent study (BEAT-ROP, ClinicalTrials.gov Identifier: NCT01232777) (n = 150), infants with stage 3+ ROP were given bilateral intravitreal injections of 0.625 mg of Avastin® bilaterally (6) resulting in a dose equal to that given to the adult macaques. As the blood retinal barrier is compromised in eyes with pathologic neovascularization, one may fear higher serum concentrations in these infants than in the monkeys. Regarding safety, the authors of this study concluded that 2800 infants were needed to assess mortality and an even larger sample for local or systemic toxicity and that the study was too small to address the question of whether intravitreal bevacizumab is safe. Thus, no attempts to monitor adverse effects were made, and serum concentrations of bevacizumab were not reported.

## Propranolol for ROP

Propranolol has been found to be efficient in reducing the growth of infantile hemangiomas in a number of patients (8,9), although no controlled trials of safety and efficacy have been reported yet. In a mouse model of ROP, propranolol was protective against retinal angiogenesis and ameliorated blood–retina barrier dysfunction in oxygen-induced retinopathy (OIR) (10).

The hypothesis of the PROP-ROP study is that in preterm infants with ROP, VEGF overexpression could be induced by beta2-adrenoreceptor stimulation and that propranolol, administered when ROP stage 2 is detected in zone II or III, could reduce the progression of the disease. As mentioned by the authors, most cases of ROP stage 2 regress spontaneously, which means that most of the infants treated with propranolol never risked blindness anyway.

Propranolol is reported to be well tolerated in most cases. However, hemodynamic effects such as bradycardia and hypotension as well as metabolic effects such as hypoglycemia may be serious (11–13). In addition, beta blockers may cause masking of hypoglycemia, insulin resistance and dyslipidemia (14). It is well known that very preterm infants already have an increased risk of hypotension (15) and deranged glucose metabolism including insulin resistance (16).

Less is known about the roles of the adrenergic system and the beta-adrenergic receptors on the development of the premature infant's brain. In rats, OIR is induced by exposure to increased oxygen concentrations from birth to postnatal day 11 followed by room air for 7 days after which proliferative retinopathy, similar to human stage 3 ROP, is found (17). In the rat brain, the timing of noradrenergic cortical innervations coincides with neurogenesis, neuronal migration, sprouting of cellular processes and the formation of synaptic contacts and occurs mainly during the first 3 weeks of postnatal life (18), just like the development of OIR and the transition to proliferative disease. Noradrenalin is a neurotransmitter that is essential for the modulation of memory (19) and for the plasticity of visual (20,21) and olfactory systems (19). Propranolol passes the blood–brain barrier and causes memory loss in chicks (19) and blocks early olfactory learning in rats (22).

It is likely that during the period when pathologic angiogenesis is a threat to the eye, windows of susceptibility occur in other parts of the central nervous system, where adequate angiogenesis and development of neural and other tissues are essential for later normal function. Administering potent blockers of VEGF and of the adrenergic system during these periods may be deleterious. As long as we lack methods to explore how the growth and modulation of neuronal networks are orchestrated, medications that possibly alter these processes for the rest of life should be avoided. In preterm infants prone to abnormal brain development, it will be impossible to sort out adverse effects of these drugs. Laser ablation of the retina is not ideal, but its effects are restricted to the eye and it usually works well. Antiangiogenetic treatment might be indicated in serious cases when laser treatment has failed, but that is different from it being an alternative to laser. There might be a place for propranolol for pathologic vessels just like for severe hemangiomas, but its use as prophylaxis to prevent proliferative ROP during stage 2 like in the PROP-ROP study should be questioned. If Avastin® is further used in infants, monitoring of serum bevacizumab and exploration of its pharmacokinetics and effects on premature babies are needed.
